# Childhood febrile illness and the risk of myopia in UK Biobank participants

**DOI:** 10.1038/eye.2016.7

**Published:** 2016-02-05

**Authors:** J A Guggenheim, C Williams, the UK Biobank Eye and Vision Consortium

**Affiliations:** 1 School of Optometry and Vision Sciences, Cardiff University, Cardiff, UK; 2 School of Social and Community Medicine, University of Bristol, Bristol, UK

## Abstract

**Purpose:**

Historical reports suggest febrile illness during childhood is a risk factor for myopia. The establishment of the UK Biobank provided a unique opportunity to investigate this relationship.

**Patients and methods:**

We studied a sample of UK Biobank participants of White ethnicity aged 40–69 years old who underwent autorefraction (*N*=91 592) and were classified as myopic (≤−0.75 Dioptres (D)), highly myopic (≤−6.00 D), or non-myopic (>−0.75 D). Self-reported age at diagnosis of past medical conditions was ascertained during an interview with a nurse at a Biobank assessment centre. Logistic regression analysis was used to calculate the odds ratio (OR) for myopia or high myopia associated with a diagnosis before age 17 years of each of nine febrile illnesses, after adjusting for potential confounders (age, sex, highest educational qualification, and birth order).

**Results:**

Rubella, mumps, and pertussis were associated with myopia: rubella, OR=1.38, 95% CI: 1.03–1.85, *P*=0.030; mumps, OR=1.32, 95% CI: 1.07–1.64, *P*=0.010; and pertussis, OR=1.39, 95% CI 1.03–1.87, *P*=0.029. Measles, rubella, and pertussis were associated with high myopia: measles, OR=1.48, 95% CI: 1.07–2.07, *P*=0.019; rubella, OR=1.94, 95% CI: 1.12–3.35, *P*=0.017; and pertussis, OR=2.15, 95% CI: 1.24–3.71, *P*=0.006. The evidence did not support an interaction between education and febrile illness in explaining the above risks.

**Conclusion:**

A history of childhood measles, rubella, or pertussis was associated with high myopia, whereas a history of childhood rubella, mumps, or pertussis was associated with any myopia. The reasons for these associations are unclear.

## Introduction

Myopia is a multifactorial disorder, with risk factors that include specific genetic variants,^1, 2, 3^ prolonged nearwork,^4, 5^ time spent outdoors,^6, 7^ maternal age,^8, 9^ and birth order.^9, 10^ Rare genetic and environmental causes of severe myopia have also been documented.^11, 12, 13, 14, 15, 16^ Nevertheless, most of the variance of refractive error in the population remains unaccounted for, and thus additional risk factors for myopia are likely to exist.^17, 18, 19, 20^

Historically, childhood febrile illness has been proposed as a predisposing factor for myopia development. For instance, Duke Elder^21^ states, ‘It has long been observed that myopia has a habit of appearing or increasing in periods of ill-health or after disease: the common belief that it starts in youth with measles or some such childish febrile illness is not without truth.' However, apart from a small study by Hirsch^22^ examining the age of contracting measles in myopic *vs* non-myopic school children, little research into this question has been carried out in recent decades. We examined this question in participants participating in the UK Biobank project.

## Materials and methods

### Participants

The UK Biobank recruited 502 649 subjects aged 37–73 years, during 2006–2010. Participants attended 1 of 22 assessment centres located in England, Scotland, or Wales, at which they completed a touch-key questionnaire, had a face-to-face interview with a trained nurse, and underwent anthropomorphic and other assessments. Later stages of the recruitment process included an ophthalmic component. All assessments adhered to standardised protocols. Ethical approval was obtained from the National Health Service (NHS) National Research Ethics Service (Ref. 11/NW/0382) and all participants provided informed consent.

Febrile illness history was ascertained during the face-to-face interview, when participants self-reported cancer and non-cancer illnesses, including the date of diagnosis by a doctor. The available illness response terms included pneumonia, encephalitis, meningitis, rheumatic fever, measles, rubella, mumps, diphtheria, and pertussis. Ethnicity, educational/professional qualifications, and birth order were recorded during the touch-key questionnaire session. For participants who underwent the ophthalmic assessment, refractive error in each eye was measured by non-cycloplegic autorefraction using a Tomey RC5000 autorefractor (Tomey GmbH, Erlangen, Germany) after participants removed their habitual spectacles or contact lenses.

### Classification of variables

Participants were classified as affected if they self-reported a diagnosis of the febrile illnesses before the age of 17 years (this age threshold having been chosen as encompassing the period of childhood when myopia most often develops^23^). Ethnicity was classified as either ‘White' (self-report of British, Irish, or any other white background) or ‘Other' (self-report of Indian, Pakistani, African, Chinese, mixed-race, or ‘prefer not to answer'). Birth order was calculated as one plus the number of older siblings, or set as missing if the number of older siblings reported was greater than the total number of siblings reported. Birth orders of 4 and above were combined into a single group, because of small numbers. The Biobank touch-key questionnaire categorized highest educational or professional qualification into seven groups: College or University degree; A-levels/AS-levels; O-levels; CSEs or equivalent; NVQ or HND or HNC or equivalent; other professional qualifications, for example: nursing, teaching; none. This scheme was reduced to four categories—(1) none; (2) O-levels or CSEs; (3) A-levels, NVQ, HND, HNC, or other professional qualification; (4) degree—which were chosen to reflect approximately equal years of academic education. Autorefractor data for participants were excluded from further analysis if the instrument labelled the reading as ‘low reliability' or ‘lower reliability'. The refractive error of a participant was taken as the average spherical equivalent (spherical power plus half the cylinder power) of their fellow eyes. If data were only available for one eye, then the spherical equivalent for that eye was used. Participants with a refractive error ≤−0.75 and ≤−6.00 D were classified as myopic and highly myopic, respectively.^8^

### Statistical analysis

Participants aged <40 years, older than 69 years, or who reported non-White ethnicity were excluded, as the numbers reporting a febrile illness were very low in age groups or ethnic groups outside this range. Those reporting a history of cataract, cataract surgery, corneal graft surgery, laser eye surgery, serious eye trauma, or having undergone retinal/vitrectomy surgery were also excluded. For each febrile illness in turn, logistic regression was used to examine the association between affection status (independent variable) and myopia (dependent variable) or high myopia (dependent variable). For the analyses of high myopia, participants with mild/moderate myopia (>−6.00 and ≤−0.75 D) were excluded. Univariate analyses were followed by multivariate analyses that included the potential confounders, age, sex, birth order, and highest educational qualification. Initially, logistic regression analyses were carried out with the *glm* function of R,^24^ separately for 10 age bins of interval 3 years (40–42, 43–45, ... 67–69 years) and the resulting log odds ratios combined using the *rma* random-effects meta-analysis function from the R *metafor* package.^25^ Analyses were carried out for the entire sample aged 40–69 years old using age as a categorical variable with 10 levels, each corresponding to a 3-year age bin.

## Results

### Participant demographics

Of the 502 656 individuals whose data were released for analysis, 114 741 (22.8%) had autorefractor readings for at least one eye. Participants were excluded if they were outside the age range 40–69 years (*N*=602), were of non-White ethnicity (*N*=12 588), or reported a history of cataract or other eye disorder (*N*=8220). Covariate information (birth order, highest educational qualification, or age at onset of febrile illness) was missing for 1739 (1.9%) of the participants, leaving 91 592 available for analysis. The mean±s.d. age was 56.9±7.9 years, the prevalence of myopia and high myopia was 30.3% and 3.9%, respectively, and the median (interquartile range) of refractive error was 0.14 D (−1.23 to 1.12 D). Table 1 presents the demographic characteristics of the study sample.

### Age dependence of illnesses

**Figure 1:**
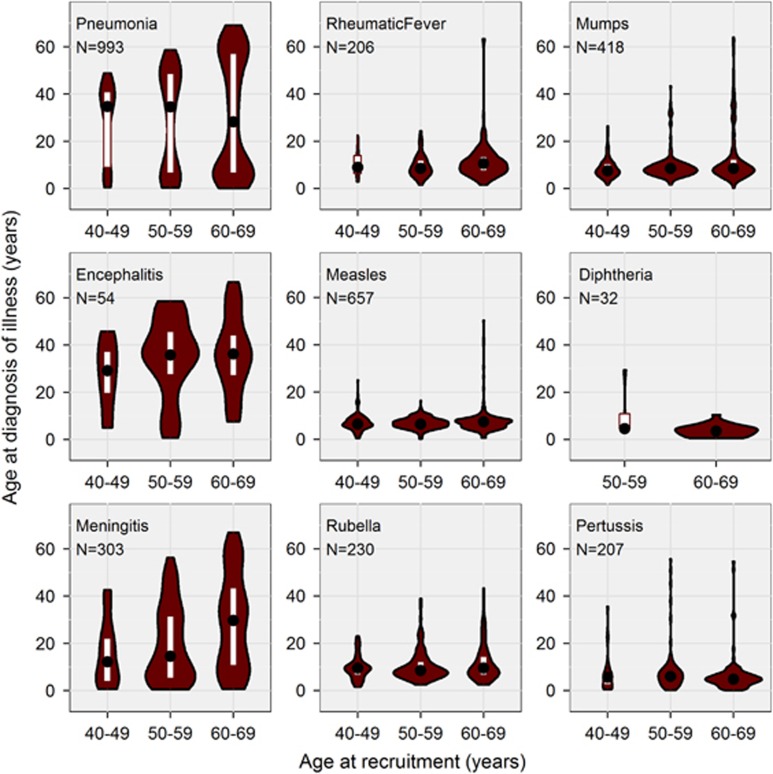
Violin plots showing age at diagnosis of febrile illnesses as a function of age at recruitment. Each panel shows the median (black circle), interquartile range (white rectangle), and the frequency distribution (smoothed histogram with brown shading, mirrored vertically; width proportional to number of affected participants in that age caetgory) of the age at diagnosis, by category of age at recruitment. The total number of cases across all three age categories is indicated (*N*). The analysis was restricted to White participants aged 40–69 rears with valid autorefraction information (*N*=91 592).

**Figure 2:**
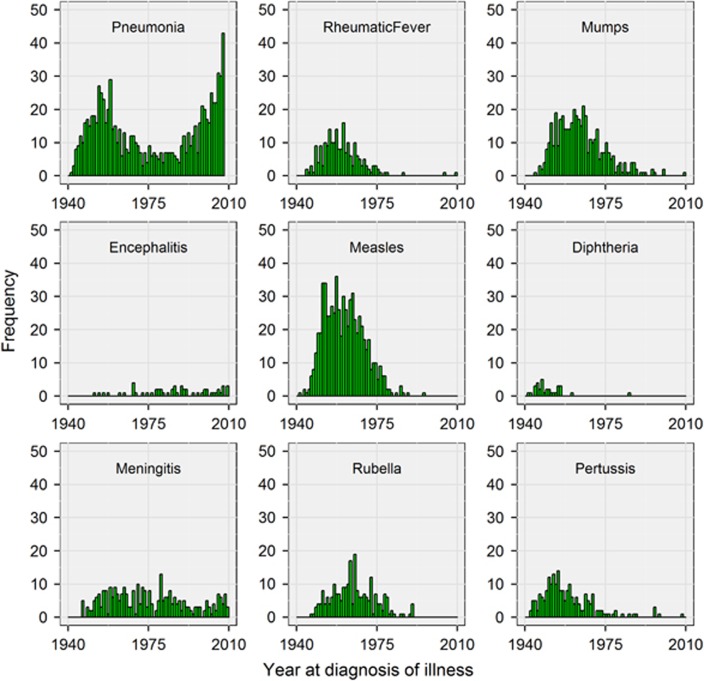
Histograms showing the year in which participants self-reported a diagnosis of febrile illness. Year at diagnosis was calculated as year of birth+age at diagnosis.

The nine febrile illnesses showed varying patterns of self-reported age at diagnosis (Figure 1). With the exception of encephalitis, the illnesses exhibited a peak onset during childhood. Pneumonia and meningitis were notable in showing secondary peaks in older and middle age, respectively. Again with the exception of encephalitis, there was a trend towards a reducing prevalence of each illness in participants born in more recent decades (Figure 2). The number of participants affected varied markedly between illnesses (Figure 1 and Table 1).

### Association between febrile illness and myopia

In an attempt to limit any excessive influence from isolated epidemic outbreaks, analyses were initially conducted separately for each of 10 age strata (40–42, 43–45, 46–48, ... 67–69 years) and the results combined using a random-effects meta-analysis.^26^ This approach was designed to downweight associations occurring only sporadically, for example, during a disease epidemic that affected individuals in one particular year, compared with associations that occurred consistently across age strata. However, no evidence of heterogeneity across age strata was found (*P*≥0.25 for Cochrane's *Q*-test, for all illnesses). In further support of consistency across age strata, the meta-analysis odds ratios were found to be similar to those for analyses of the full 40–69 years age spectrum. Hence, only the latter results are reported (Table 2).

*Pneumonia, meningitis, and rheumatic fever*: There was no indication that any of these three febrile illnesses was associated with myopia.

*Measles*: Before adjustment for potential confounders, measles had a modest, positive association with myopia (OR=1.27, 95% CI: 1.08–1.50, *P*=0.003). However, adjusting for confounders reduced the strength and magnitude of the association (OR=1.14, *P*=0.12).

*Rubella*: A larger, positive association (OR=1.55, *P*=0.002) was observed between rubella and myopia, which was moderately attenuated after adjusting for potential confounders (OR=1.38, 95% CI: 1.03−1.84, *P*=0.030).

*Mumps*: Mumps showed a similar pattern of association with myopia to that of rubella (unadjusted OR=1.50, *P*<0.001; adjusted OR=1.32, 95% CI: 1.07−1.64, *P*=0.010).

*Pertussis*: Before adjusting for potential confounders, there was a modest positive association between pertussis and myopia (OR=1.40, 95% CI: 1.05–1.87, *P*=0.023), which was not attenuated in the adjusted analysis (OR=1.39, 95% CI: 1.03–1.87, *P*=0.029).

*Encephalitis* and *diphtheria*: There were too few cases of encephalitis and diphtheria to obtain reliable risk estimates.

### Association between febrile illness and high myopia

*Pneumonia, rheumatic fever, and mumps*: These three febrile illnesses were not convincingly associated with high myopia, although there was suggestive evidence of an association with mumps in the unadjusted analysis (OR=1.59, 95% CI: 1.00–2.51, *P*=0.049) (Table 3).

*Measles*: The evidence linking measles to high myopia was stronger than that linking it to any level of myopia. A moderate positive association was observed between high myopia and measles before adjustment for potential confounders (OR =1.71, *P*=0.001); adjustment for potential confounders partially reduced the estimated effect size (OR=1.48, 95% CI: 1.07–2.07, *P*=0.019).

*Rubella and Pertussis*: There was support for an association between rubella and high myopia (adjusted OR=1.94, 95% CI: 1.12–3.35, *P*=0.017) and between pertussis and high myopia (adjusted OR=2.15, 95% CI: 1.24–3.71, *P*=0.006). These estimates were similar to those prior to adjustment for potential confounders (Table 3).

*Encephalitis, meningitis, and diphtheria*: There were too few participants diagnosed with these illnesses to calculate reliable risk estimates.

## Discussion

In White UK Biobank participants aged 40–69 years, a self-reported history of rubella, mumps, or pertussis during childhood was associated with an ~30% increased risk of myopia in adulthood. A history of measles, rubella, or pertussis was associated with a 50–110% increased risk of high myopia.

The mechanism previously proposed to explain a causal association between febrile illness and myopia is a change in the biomechanical properties of the sclera after the illness.^21^ Intriguingly, measles, mumps, and rubella are all single-strand RNA viruses that have very high mutation rates compared with DNA viruses; therefore, the immunological and inflammatory responses to these infective agents may be relevant to their association with myopia. In contrast, *Bordetella pertussis*, the Gram-negative bacterium responsible for pertussis secretes a range of toxins, one of which—adenylate cyclase toxin—increases levels of intracellular cAMP in host cells, which could conceivably be related to myopia susceptibility through a cAMP-dependent mechanism.^27, 28, 29^ Alternatively, and more generally, it also seems plausible that children recovering from a febrile illness might spend prolonged periods of time indoors and reading, compared with their unaffected peers, both of which have been associated with incident myopia.^7^ However, it is unclear why such an effect would occur for certain febrile illnesses yet not for others. Other potential explanations for the observed associations between febrile illnesses and myopia are reverse causality and confounding.

Reverse causality, that is children with myopia having an increased risk of developing a febrile illness, seems plausible: for instance, myopic children have been reported to spend less time playing outdoors^30^ and being indoors for longer than average may increase a child's risk of infection. However, if true, this relationship would again be expected to confer a higher risk of all communicable illnesses, not just those found to be associated here. Of the potential confounders examined, age, highest educational qualification, and birth order were all strongly correlated with myopia (all *P*<0.001). Furthermore, highest educational qualification was associated with self-reported history of measles, rubella, and mumps (*X*^2^ test; all *P*<0.001), although not pneumonia and pertussis. These interrelationships reflect the wide age span of the UK Biobank participants, along with increased myopia prevalence, years spent in education, and reduced prevalence of febrile illnesses in younger generations. Confounding because of unmeasured variables thus appears feasible. The risk of myopia or high myopia associated with a history of febrile illness varied across age strata and to a greater extent across educational strata (Supplementary Tables S1–S6). The difference in effect size across educational qualification strata was most apparent for mumps in relation to the risk of myopia, and for rubella in relation to the risk of high myopia. For instance, the OR for myopia associated with a history of mumps varied from OR=1.17 (95% CI: 0.87–1.57) for those in the top educational qualification category, to OR=1.90 (95% CI: 1.26–2.88) for those in the second-highest category (Supplementary Table S1). Similarly, the OR for high myopia associated with a history of rubella varied from OR=1.29 (95% CI: 0.55–3.03) to OR=4.95 (95% CI: 2.26–10.86) for those in the top and second-highest educational strata, respectively (Supplementary Table S3). To formally examine the presence of confounding, we tested for an age × febrile illness interaction, or an educational qualification × febrile illness interaction. However these tests did not support the presence of an interaction. Under- or over-reporting of a childhood febrile illness in more highly educated participants may have contributed to the observed differences in effect size across educational strata. However, arguing against this cause, the pattern of effect size across educational followed an inverted U-shape, that is, the risk of myopia or high myopia associated with febrile illness was greatest in participants with an intermediate educational qualification (specifically, the second-highest category) rather than in the highest or lowest category.

### Strengths, weaknesses, and limitations of the study

This study benefitted from a large sample size and standardised, systematic methods of data collection. Weaknesses were that the febrile illnesses were self-reported—usually decades after their onset—rather than being collected from medical records, that no attempt was made to validate the sensitivity and specificity of the febrile illness self-reports, the large age span of the sample (which increased the risk of confounding effects), and the inability of the study design to distinguish causal from non-causal associations.

The non-random nature of the Biobank recruitment process means that these results may not generalise to the whole UK population. In addition, the rarity of most febrile illnesses in countries that are currently experiencing a high incidence of myopia makes it unlikely that febrile illness is an important contributor to the current myopia epidemic.

In summary, the findings of this study support a previously reported association between measles and high myopia^22^ and further suggest that childhood rubella, mumps, and pertussis are also associated with myopia and/or high myopia. There was no evidence that meningitis or rheumatic fever were associated with myopia. Further work will be required to discover the causal relationships underlying these associations.


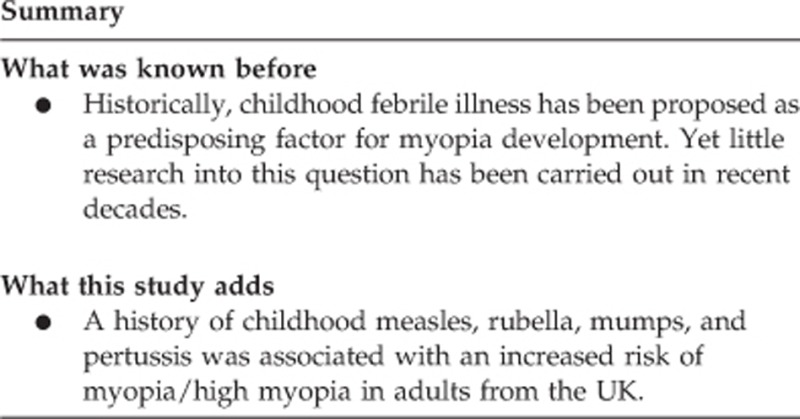


## Figures and Tables

**Table 1:** Subjects demographics

*Variable*	*Group*	N *(%)*	N *(%)*
Myopia	Myopic	27 752 (30.3%)	
	Non-myopic	63 840 (69.7%)	
			
High myopia	Highly myopic	3586 (5.3%)	
	Non-myopic	63 840 (94.7%)	
			
Sex	Male	42 039 (45.9%)	
	Female	49 553 (54.1%)	
			
Ethnicity	White	91 592 (100.0%)	
			
Birth order	1	43 009 (47.0%)	
	2	27 779 (30.3%)	
	3	11 663 (12.7%)	
	4+	9141 (10.0%)	
			
Highest qualification	University degree	32 048 (35.0%)	
	A-levels or similar^a^	21 226 (23.2%)	
	O-levels or CSEs	24 774 (27.0%)	
	None	13 544 (14.8%)	
			
Febrile illness	Age at onset	Any age	Before 17 years
	Pneumonia	993 (1.1%)	400 (0.4%)
	Encephalitis	54 (0.1%)	10 (0.0%)
	Meningitis	303 (0.3%)	142 (0.2%)
	Rheumatic fever	206 (0.2%)	183 (0.2%)
	Measles	657 (0.7%)	649 (0.7%)
	Rubella	230 (0.3%)	196 (0.2%)
	Mumps	418 (0.5%)	371 (0.4%)
	Diphtheria	32 (0.0%)	31 (0.0%)
	Pertussis	207 (0.2%)	193 (0.2%)

a Includes NVQ, HND, HNC, and other professional qualifications.

**Table 2:** Association between febrile illness before age 17 years and myopia (*N*=91 592)

*Illness*	*Univariate analysis*		P*-value*	*Multivariate analysis*^a^		P*-value*
	*OR*	*95% CI*		*OR*	*95% CI*	
Pneumonia	1.083	0.877–1.337	0.458	1.157	0.933–1.434	0.184
Meningitis	0.840	0.579–1.219	0.359	0.808	0.554–1.179	0.269
Rheumatic fever	1.014	0.740–1.390	0.929	1.148	0.832–1.584	0.401
Measles	1.274	1.084–1.496	0.003	1.139	0.966–1.342	0.121
Rubella	1.555	1.168–2.069	0.002	1.380	1.033–1.845	0.030
Mumps	1.495	1.214–1.842	<0.001	1.322	1.069–1.635	0.010
Pertussis	1.400	1.047–1.874	0.023	1.392	1.034–1.874	0.029

a Adjusted for age, sex, highest educational qualification, and birth order.

**Table 3:** Association between febrile illness before age 17 years and high myopia (*N*=67 426)

*Illness*	*Univariate analysis*		P*-value*	*Multivariate analysis*^a^		P*-value*
	*OR*	*95% CI*		*OR*	*95% CI*	
Pneumonia	1.377	0.882–2.149	0.159	1.551	0.987–2.436	0.057
Rheumatic fever	0.700	0.287–1.712	0.435	0.835	0.339–2.059	0.696
Measles	1.712	1.235–2.372	0.001	1.484	1.067–2.065	0.019
Rubella	2.288	1.335–3.920	0.003	1.941	1.124–3.349	0.017
Mumps	1.586	1.002–2.508	0.049	1.325	0.834–2.107	0.234
Pertussis	2.230	1.303–3.819	0.003	2.147	1.242–3.711	0.006

a Adjusted for age, sex, highest educational qualification, and birth order.
