# Supplementary material for: Childhood febrile illness and the risk of myopia in UK Biobank participants
Source: Eye (Lond). 2016 Feb 5;30(4):608–14. doi: 10.1038/eye.2016.7 (PMC4834038; doi:10.1038/eye.2016.7)
Supplement: Supplementary Tables [file eye20167x1.doc]

**Supplementary Information**

**Table S1. Association between febrile illness to age 17 and myopia: Analyses stratified by highest educational qualification.** Age, sex and birth order were included in all models.

|  | **None (N=13,544)** | | | | **O-level/CSE (N=24,774)** | | | | **A-level/equiv. (N=21,226)** | | | | **Degree (N=32,048)** | | | |
| --- | --- | --- | --- | --- | --- | --- | --- | --- | --- | --- | --- | --- | --- | --- | --- | --- |
|  | **OR** | **95% CI** | **P** | **Rate** | **OR** | **95% CI** | **P** | **Rate** | **OR** | **95% CI** | **P** | **Rate** | **OR** | **95% CI** | **P** | **Rate** |
| Pneumonia | 0.835 | (0.412~1.690) | 0.616 | 0.50% | 1.191 | (0.763~1.858) | 0.442 | 0.40% | 0.995 | (0.638~1.553) | 0.982 | 0.50% | 1.379 | (0.986~1.931) | 0.061 | 0.40% |
| Measles | 0.547 | (0.217~1.380) | 0.201 | 0.40% | 1.082 | (0.761~1.538) | 0.662 | 0.60% | 1.347 | (0.953~1.904) | 0.092 | 0.70% | 1.151 | (0.915~1.449) | 0.229 | 0.90% |
| Rubella | NA | NA | NA | 0.10% | 1.474 | (0.837~2.597) | 0.179 | 0.20% | 1.673 | (0.978~2.863) | 0.060 | 0.30% | 1.152 | (0.739~1.797) | 0.532 | 0.20% |
| Mumps | NA | NA | NA | 0.20% | 1.234 | (0.759~2.005) | 0.397 | 0.30% | 1.902 | (1.256~2.880) | 0.002 | 0.40% | 1.165 | (0.863~1.572) | 0.318 | 0.50% |
| Pertussis | NA | NA | NA | 0.20% | 1.849 | (1.060~3.224) | 0.030 | 0.20% | 1.442 | (0.726~2.861) | 0.296 | 0.20% | 1.274 | (0.819~1.980) | 0.282 | 0.30% |

NA=Too few febrile illness cases (N<5) to reliably determine risk. Rate=Prevalence of febrile illness within highest qualification stratum (%).

**Table S2. Cross-tabulation of febrile illness prior to age 17 and myopia, stratified by highest educational qualification**.

| Refractive | **Pneumonia** | **Pneumonia** | **Measles** | **Measles** | **Rubella** | **Rubella** | **Mumps** | **Mumps** | **Pertussis** | **Pertussis** | **Education** |
| --- | --- | --- | --- | --- | --- | --- | --- | --- | --- | --- | --- |
| error | **Controls** | **Cases** | **Controls** | **Cases** | **Controls** | **Cases** | **Controls** | **Cases** | **Controls** | **Cases** | **stratum** |
| Non-myopic | 11369 (84%) | 59 (87%) | 11381 (84%) | 47 (90%) | 11422 (84%) | 6 (75%) | 11405 (84%) | 23 (85%) | 11407 (84%) | 21 (88%) | None |
| Myopic | 2107 (16%) | 9 (13%) | 2111 (16%) | 5 (10%) | 2114 (16%) | 2 (25%) | 2112 (16%) | 4 (15%) | 2113 (16%) | 3 (12%) | (N=13,544) |
| Non-myopic | 17960 (73%) | 65 (70%) | 17916 (73%) | 109 (71%) | 17992 (73%) | 33 (63%) | 17973 (73%) | 52 (68%) | 17994 (73%) | 31 (60%) | O-levels/CSEs |
| Myopic | 6721 (27%) | 28 (30%) | 6705 (27%) | 44 (29%) | 6730 (27%) | 19 (37%) | 6725 (27%) | 24 (32%) | 6728 (27%) | 21 (40%) | (N=24,774) |
| Non-myopic | 14992 (71%) | 73 (73%) | 14974 (71%) | 91 (64%) | 15032 (71%) | 33 (59%) | 15014 (71%) | 51 (55%) | 15042 (71%) | 23 (64%) | A-levels |
| Myopic | 6134 (29%) | 27 (27%) | 6110 (29%) | 51 (36%) | 6138 (29%) | 23 (41%) | 6120 (29%) | 41 (45%) | 6148 (29%) | 13 (36%) | (N=21,226) |
| Non-myopic | 19247 (60%) | 75 (54%) | 19151 (60%) | 171 (57%) | 19277 (60%) | 45 (56%) | 19223 (60%) | 99 (56%) | 19277 (60%) | 45 (56%) | Degree |
| Myopic | 12662 (40%) | 64 (46%) | 12595 (40%) | 131 (43%) | 12691 (40%) | 35 (44%) | 12649 (40%) | 77 (44%) | 12690 (40%) | 36 (44%) | (N=32,048) |

Percentages show prevalence of myopia within disease affectation (case/control) group.

**Table S3. Association between febrile illness prior to age 17 and high myopia: Analyses stratified by highest educational qualification.** Age, sex and birth order were included in all models.

|  | **None (N=11,605)** | | | | | | **O-level/CSE (N=18,771)** | | | | **A-level/equiv. (N=15,781)** | | | | **Degree (N=21,269)** | | | |
| --- | --- | --- | --- | --- | --- | --- | --- | --- | --- | --- | --- | --- | --- | --- | --- | --- | --- | --- |
|  | **OR** | **95% CI** | | | **P** | **Rate** | **OR** | **95% CI** | **P** | **Rate** | **OR** | **95% CI** | **P** | **Rate** | **OR** | **95% CI** | **P** | **Rate** |
| Pneumonia | NA | NA | NA | 0.50% | | | NA | NA | NA | 0.40% | 2.711 | (1.294~5.679) | 0.008 | 0.50% | 1.593 | (0.841~3.015) | 0.153 | 0.40% |
| Measles | NA | NA | NA | 0.40% | | | 1.28 | (0.560~2.927) | 0.559 | 0.60% | 2.283 | (1.178~4.425) | 0.014 | 0.60% | 1.337 | (0.862~2.075) | 0.195 | 0.90% |
| Rubella | NA | NA | NA | 0.10% | | | NA | NA | NA | 0.20% | 4.948 | (2.255~10.858) | <0.001 | 0.30% | 1.286 | (0.546~3.027) | 0.565 | 0.20% |
| Mumps | NA | NA | NA | 0.20% | | | NA | NA | NA | 0.30% | 1.994 | (0.788~5.043) | 0.145 | 0.40% | 1.283 | (0.717~2.297) | 0.402 | 0.50% |
| Pertussis | NA | NA | NA | 0.20% | | | NA | NA | NA | 0.20% | 5.049 | (1.900~13.420) | 0.001 | 0.20% | 1.827 | (0.856~3.898) | 0.119 | 0.20% |

NA=Too few febrile illness cases (N<5) to reliably determine risk. Rate=Prevalence of febrile illness within highest qualification stratum (%).

**Table S4. Cross-tabulation of febrile illness** prior to age 17 and high myopia, stratified by highest educational qualification.

| Refractive | **Pneumonia** | **Pneumonia** | **Measles** | **Measles** | **Rubella** | **Rubella** | **Mumps** | **Mumps** | **Pertussis** | **Pertussis** | **Education** |
| --- | --- | --- | --- | --- | --- | --- | --- | --- | --- | --- | --- |
| error | **Controls** | **Cases** | **Controls** | **Cases** | **Controls** | **Cases** | **Controls** | **Cases** | **Controls** | **Cases** | **stratum** |
| Non-myopic | 11369 (98%) | 59 (100%) | 11381 (98%) | 47 (98%) | 11422 (98%) | 6 (100%) | 11405 (98%) | 23 (100%) | 11407 (98%) | 21 (100%) | None |
| Highly myopic | 177 (2%) | 0 (0%) | 176 (2%) | 1 (2%) | 177 (2%) | 0 (0%) | 177 (2%) | 0 (0%) | 177 (2%) | 0 (0%) | (N=11,605) |
| Non-myopic | 17960 (96%) | 65 (97%) | 17916 (96%) | 109 (95%) | 17992 (96%) | 33 (97%) | 17973 (96%) | 52 (96%) | 17994 (96%) | 31 (94%) | O-levels/CSEs |
| Highly myopic | 744 (4%) | 2 (3%) | 740 (4%) | 6 (5%) | 745 (4%) | 1 (3%) | 744 (4%) | 2 (4%) | 744 (4%) | 2 (6%) | (N=18,771) |
| Non-myopic | 14992 (95%) | 73 (90%) | 14974 (95%) | 91 (90%) | 15032 (96%) | 33 (80%) | 15014 (95%) | 51 (91%) | 15042 (95%) | 23 (82%) | A-levels |
| Highly myopic | 708 (5%) | 8 (10%) | 706 (5%) | 10 (10%) | 708 (4%) | 8 (20%) | 711 (5%) | 5 (9%) | 711 (5%) | 5 (18%) | (N=15,781) |
| Non-myopic | 19247 (91%) | 75 (87%) | 19151 (91%) | 171 (88%) | 19277 (91%) | 45 (88%) | 19223 (91%) | 99 ( 88%) | 19277 (91%) | 45 (85%) | Degree |
| Highly myopic | 1936 (9%) | 11 (13%) | 1924 (9%) | 23 (12%) | 1941 (9%) | 6 (12%) | 1934 (9%) | 13 (12%) | 1939 (9%) | 8 (15%) | (N=21,269) |

Percentages show prevalence of high myopia within disease affectation (case/control) group.

**Table S5. Cross-tabulation of febrile illness prior to age 17 and myopia, stratified by age decade.**

| Refractive | **Pneumonia** | **Pneumonia** | **Measles** | **Measles** | **Rubella** | **Rubella** | **Mumps** | **Mumps** | **Pertussis** | **Pertussis** | **Age** |
| --- | --- | --- | --- | --- | --- | --- | --- | --- | --- | --- | --- |
| error | **Controls** | **Cases** | **Controls** | **Cases** | **Controls** | **Cases** | **Controls** | **Cases** | **Controls** | **Cases** | **decade** |
| Non-myopic | 12917 (66%) | 22 (50%) | 12847 (66%) | 92 (65%) | 12914 (66%) | 25 (58%) | 12890 (66%) | 49 (63%) | 12922 (66%) | 17 (65%) | 40-49 |
| Myopic | 6786 (34%) | 22 (50%) | 6759 (34%) | 49 (35%) | 6790 (34%) | 18 (42%) | 6779 (34%) | 29 (37%) | 6799 (34%) | 9 (35%) |  |
| Non-myopic | 19664 (66%) | 73 (61%) | 19593 (66%) | 144 (56%) | 19690 (66%) | 47 (53%) | 19657 (66%) | 80 (53%) | 19701 (66%) | 36 (55%) | 50-59 |
| Myopic | 10226 (34%) | 46 (39%) | 10161 (34%) | 111 (44%) | 10231 (34%) | 41 (47%) | 10200 (34%) | 72 (47%) | 10242 (34%) | 30 (45%) |  |
| Non-myopic | 30987 (74%) | 177 (75%) | 30982 (75%) | 182 (72%) | 31119 (74%) | 45 (69%) | 31068 (75%) | 96 (68%) | 31097 (75%) | 67 (66%) | 60-69 |
| Myopic | 10612 (26%) | 60 (25%) | 10601 (25%) | 71 (28%) | 10652 (26%) | 20 (31%) | 10627 (25%) | 45 (32%) | 10638 (25%) | 34 (34%) |  |

**Table S6. Cross-tabulation of febrile illness** prior to age 17 and high myopia, stratified by age decade.

| Refractive | **Pneumonia** | **Pneumonia** | **Measles** | **Measles** | **Rubella** | **Rubella** | **Mumps** | **Mumps** | **Pertussis** | **Pertussis** | **Age** |
| --- | --- | --- | --- | --- | --- | --- | --- | --- | --- | --- | --- |
| error | **Controls** | **Cases** | **Controls** | **Cases** | **Controls** | **Cases** | **Controls** | **Cases** | **Controls** | **Cases** | **decade** |
| Non-myopic | 12917 (93%) | 22 (88%) | 12847 (93%) | 92 (94%) | 12914 (93%) | 25 (89%) | 12890 (93%) | 49 (96%) | 12922 (93%) | 17 (89%) | 40-49 |
| Highly myopic | 922 (7%) | 3 (12%) | 919 (7%) | 6 (6%) | 922 (7%) | 3 (11%) | 923 (7%) | 2 (4%) | 923 (7%) | 2 (11%) |  |
| Non-myopic | 19664 (93%) | 73 (92%) | 19593 (93%) | 144 (88%) | 19690 (93%) | 47 (89%) | 19657 (93%) | 80 (89%) | 19701 (93%) | 36 (82%) | 50-59 |
| Highly myopic | 1414 (7%) | 6 (8%) | 1400 (7%) | 20 (12%) | 1414 (7%) | 6 (11%) | 1410 (7%) | 10 (11%) | 1412 (7%) | 8 (18%) |  |
| Non-myopic | 30987 (96%) | 177 (94%) | 30982 (96%) | 182 (93%) | 31119 (96%) | 45 (88%) | 31068 (96%) | 96 (92%) | 31097 (96%) | 67 (93%) | 60-69 |
| Highly myopic | 1229 (4%) | 12 (6%) | 1227 (4%) | 14 (7%) | 1235 (4%) | 6 (12%) | 1233 (4%) | 8 (8%) | 1236 (4%) | 5 (7%) |  |
